# Clinical value of serum neuron-specific enolase in sepsis-associated encephalopathy: a systematic review and meta-analysis

**DOI:** 10.1186/s13643-024-02583-4

**Published:** 2024-07-22

**Authors:** Meiling Zhi, Jian Huang, Xuli Jin

**Affiliations:** 1 Clinical Laboratory, Hangzhou Ninth People’s Hospital, No. 98 Yilong Road, Qiantang District, Hangzhou, 310020 China; 2 Emergency Internal Medicine, Hangzhou Ninth People’s Hospital, Hangzhou, 310020 China

**Keywords:** Sepsis-associated encephalopathy, Neuron-specific enolase, Meta-analysis, Sepsis

## Abstract

**Objective:**

This study aimed to investigate the serum levels of neuron-specific enolase (NSE) in sepsis-associated encephalopathy (SAE) and perform a meta-analysis to assess the diagnostic and prognostic potential of serum NSE in SAE patients.

**Methods:**

We searched English and Chinese databases for studies related to SAE that reported serum NSE levels until November 2023. We extracted information from these studies including the first author and year of publication, the number of samples, the gender and age of patients, the collection time of blood samples in patients, the assay method of serum NSE, the study methods, and the levels of serum NSE with units of ng/mL. The quality assessment of diagnostic accuracy studies 2 (QUADAS-2) tool was used to evaluate the study quality. A meta-analysis was performed using Review Manager version 5.3, employing either a random effects model or a fixed effects model.

**Results:**

A total of 17 studies were included in the final meta-analysis, including 682 SAE patients and 946 NE patients. The meta-analysis demonstrated significantly higher serum NSE levels in SAE patients compared to NE patients (*Z* = 5.97, *P* < 0.001, MD = 7.79, 95%CI 5.23–10.34), irrespective of the method used for serum NSE detection (*Z* = 6.15, *P* < 0.001, mean difference [MD] = 7.75, 95%CI 5.28–10.22) and the study methods (*Z* = 5.97, *P* < 0.001, MD = 7.79, 95%CI 5.23–10.34). Furthermore, sepsis patients with a favorable outcome showed significantly lower levels of serum NSE compared to those with an unfavorable outcome (death or adverse neurological outcomes) (*Z* = 5.44, *P* < 0.001, MD = − 5.34, 95%CI − 7.26–3.42).

**Conclusion:**

The Serum level of NSE in SAE patients was significantly higher than that in septic patients without encephalopathy. The higher the serum NSE level in SAE patients, the higher their mortality rate and incidence of adverse neurological outcomes.

## Introduction

Sepsis is an imbalanced response to infection that ultimately leads to life-threatening organ dysfunction. Epidemiological data have shown that the global incidence of nosocomial sepsis is 189 cases per 100,000 person-years, with a case fatality rate of approximately 26.7%. Sepsis is the second leading cause of death among ICU patients worldwide [1, 2]. In China, the incidence of sepsis in the ICU is around 30%, with a case fatality rate exceeding 25%. The prognosis for patients is poor [3, 4]. Sepsis-associated encephalopathy (SAE) is characterized by diffuse cerebral dysfunction and abnormal expression of the nervous system as a secondary effect of sepsis. It is a common complication in patients with severe sepsis, with a case fatality rate of up to 70%. Survivors also experience neurological dysfunction, which significantly impacts their quality of life [5, 6]. However, due to the unclear pathogenesis of SAE and the absence of definitive biomarkers for diagnosis, SAE is currently diagnosed through exclusion criteria [7]. Therefore, studying biomarkers associated with SAE could provide a reference for diagnosis, and help to explore new therapeutic directions and find targets for the development of novel drugs.

Neuron-specific enolase (NSE) is an acidic protein widely found in nerve tissue, existing in very small quantities in serum and cerebrospinal fluid. Abnormal elevation of NSE levels in blood and cerebrospinal fluid indicates brain injury, making it a biomarker used for diagnosing brain injury, stroke, and ischemic-hypoxic encephalopathy [8, 9]. Nguyen DN et al. reported elevated serum NSE levels among patients with severe sepsis as well as septic shock, which were linked to severe brain disease and brain injury [10]. Furthermore, a prospective observational study by Zhang LN, et al. found higher serum NSE levels in SAE patients compared to septic patients without encephalopathy (NE patients) [11]. Animal experiments have also demonstrated a correlation between serum NSE levels in sepsis rats and the rate of apoptosis in the hippocampus [12]. Reducing serum NSE levels through medication has shown the potential to improve brain injury severity in sepsis animal models [13]. Therefore, these studies suggest that serum NSE could serve as a potential diagnostic biomarker for SAE.

However, due to the lack of large-scale clinical studies, serum NSE has not been included as a biomarker for the diagnosis, treatment, and prognosis prediction of brain injury in sepsis patients. To clarify the value of NSE in SAE patients, we made every effort to search for studies on NSE in SAE patients and conduct meta-analysis. In this study, our objective was to investigate the serum NSE levels and conduct a meta-analysis on its diagnostic and prognostic potential among SAE patients.

## Methods

### Search strategy

We searched for the following keywords: “sepsis-associated encephalopathy”, “septic encephalopathy”, “brain dysfunction”, “neuron-specific enolase”, “NSE”, “septic shock”, and “sepsis” in various databases, including Web of Science, PubMed, ScienceDirect, Cochrane Library, China National Knowledge Infrastructure (CNKI), WanFang, and Chongqing VIP Chinese Science and Technology Journal Database (CQVIP), until November 20, 2023. In addition, we have prospectively registered this topic in PROSPERO (https://www.crd.york.ac.uk/PROSPERO/).

### Inclusion and exclusion criteria

Inclusion criteria were as follows: (1) Studies involving patients diagnosed with sepsis or septic shock. (2) Studies involving patients with SAE, or sepsis accompanied with brain dysfunction. (3) Studies with NSE levels evaluated in serum samples.

Exclusion criteria were as follows: (1) studies simultaneously published in different databases. (2) The following types of study: animal studies, case reports, non-controlled trials, reviews, and meta-analyses. (3) Studies with NSE detected in non-serum samples. (4) Study unable to obtain complete data.

### Bias analysis

Quality assessment of diagnostic accuracy studies 2 (QUADAS-2) was used to assess the bias analysis of the study by two independent researchers. Herein, risk of bias and applicability concerns were analyzed for each study, including high, low, and unclear risk or concern.

### Data extraction and conversion

Two researchers independently extracted data from the included studies. The extracted information included the first author and year of publication, the number of samples, the gender and age of patients, the collection time of blood samples in patients, the assay method of serum NSE, the study methods (prospective, retrospective, or other), and the levels of serum NSE with units of ng/mL. Additionally, we utilized the methods of Luo D et al. [14] and Wan X et al. [15] to estimate the mean and standard deviation using the median and interquartile range.

### Statistical analysis

In the present study, a meta-analysis was performed by Review Manager version 5.3. Based on the results of the heterogeneity test, if there was a significant difference in heterogeneity (*I*^2^ > 50%, *P* < 0.05), a random-effects model (REM) was adopted for the meta-analysis. Otherwise, a fixed-effects model (FEM) was chosen. Funnel plots were utilized to visualize potential publication bias, and a significance level of *P* < 0.05 indicated a significant difference for all meta-analyses.

## Results

### Search results

**Fig. 1:**
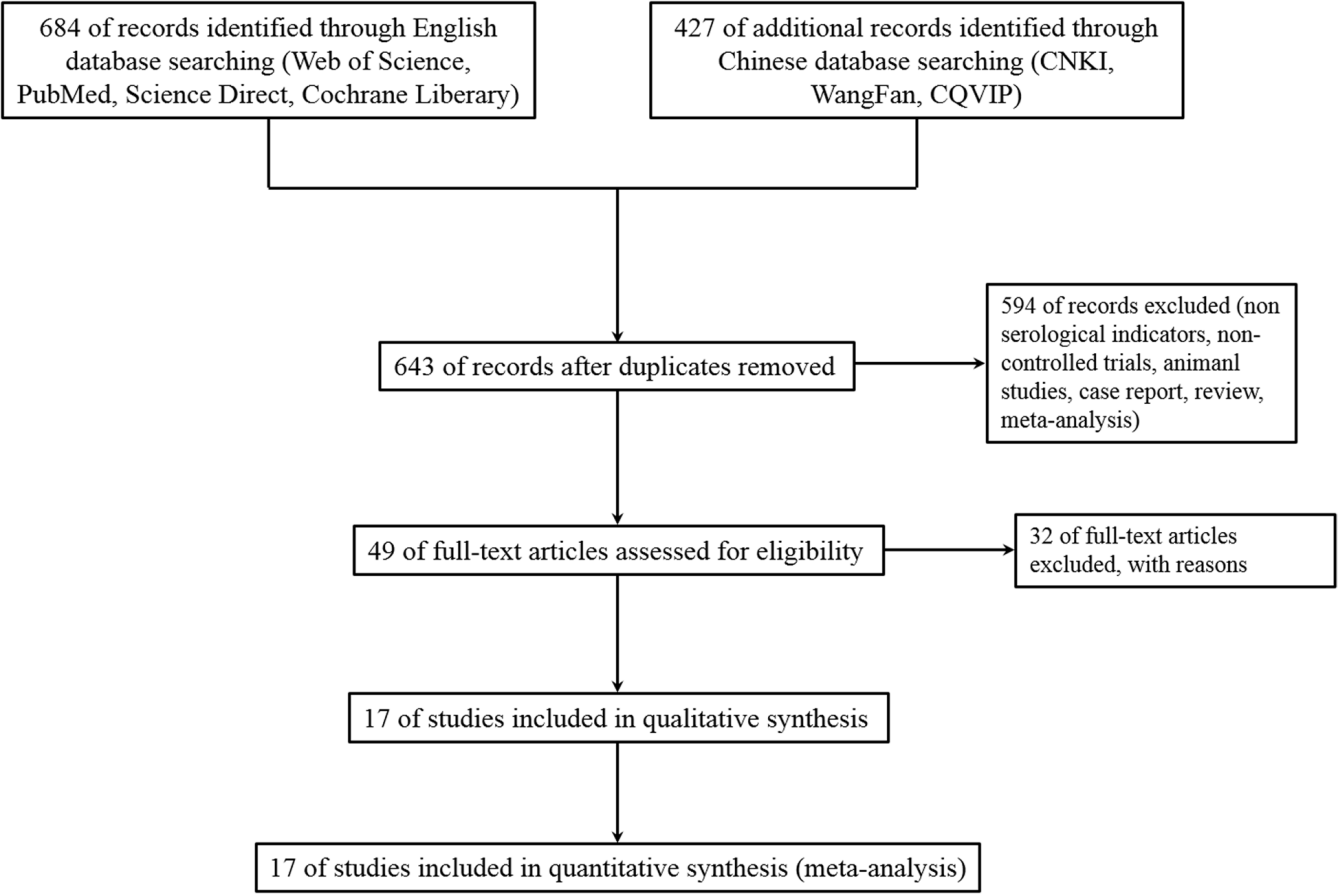
Research database retrieval process diagram

We conducted a search for specific keywords in designated databases in both English and Chinese languages. In total, 1111 relevant studies were identified, with 684 from the English database (309 from Web of Science, 246 from PubMed, 105 from Science Direct, and 24 from Cochrane Library), and 427 from the Chinese database (93 from CNKI, 237 from WangFan, and 97 from CQVIP). After removing 468 duplicate studies, we screened 643 studies based on their abstracts and titles, ultimately selecting 49 studies for full-text reading. Finally, 17 studies were finally included in the meta-analysis (Fig. 1), of which 8 were described in English (Yao B, et al. [16], Zhang LN, et al. [11], Lu CX, et al. [17], Ehler J, et al. [18], Erikson K, et al. [19], Orhun G, et al. [20], Guo W, et al. [21] and Cao ZG, et al. [22]) and 9 were described in Chinese (Feng Q, et al. [23], Li K, et al. [24], Yan S, et al. [25], Hui W, et al. [26], Zhao XK, et al. [27], Yu GL, et al. [28], Li XL, et al. [29], Xiao HT, et al. [30] and Yu DY, et al. [31]). These studies included a total of 682 SAE patients and 946 NE patients (Table 1).

**Table 1:** General characteristics of studies in the final analysis

Study (first author, year)	Sample size (*n*)		Male/female (*n*)		Age (years)		Collection time of SAE patients	Assay methods	Design
	SAE	NE	SAE	NE	SAE	NE			
Yao B, 2014 [16]	48	64	33/15	40/24	56 ± 16	52 ± 17	ICU admission	CLIA	Prospective and observational study
Zhang LN, 2016 [11]	29	28	20/9	13/15	55.55 ± 12.72	56.21 ± 12.85	Within 24 h after ICU admission	ELISA	Prospective and observational study
Lu CX, 2016 [17]	34	52	24/10	33/19	59.15 ± 8.80	55.39 ± 8.14	NA	NA	Retrospective study
Ehler J, 2019 [18]	12	0	4/8	0/0	67.8 ± 12.1	NA	NA	CLIA	Prospective, longitudinal observational study
Erikson K, 2019 [19]	10	12	4/6	10/2	62.4 (49–70.5)	61.8 (60.1–78.5)	When CAM-ICU assessed	CLIA	Prospective and observational study
Orhun G, 2019 [20]	86	0	50/36	0/0	53.2 ± 10.4	NA	Onset of acute neurological dysfunction	ELISA	Prospective and observational study
Cao ZG, 2023 [22]	35	65	15/20	31/34	58.51 ± 8.25	57.69 ± 8.66	Day of the medical visit	ELISA	NA
Feng Q, 2017 [23]	36	23	21/15	14/9	52 ± 14	57 ± 15	24 h after admission	CLIA	Retrospective study
Li K, 2019 [24]	28	102	NA	NA	NA	NA	24 h after admission	ELISA	Retrospective study
Yan S, 2019 [25]	58	94	44/14	60/34	55.8 ± 16.4	55.0 ± 18.3	Within 24 h after ICU admission	ELISA	NA
Hui W, 2020 [26]	30	30	17/13	19/11	50.5 ± 2.3	50.8 ± 2.5	24 h after admission	CLIA	Retrospective study
Zhao XK, 2020 [27]	22	78	13/9	42/33	64.7 ± 12.2	65.1 ± 11.8	When diagnosed with sepsis	ELISA	Retrospective study
Yu GL, 2020 [28]	90	90	49/41	47/43	53.61 ± 12.74	52.89 ± 11.65	NA	ELISA	NA
Guo W, 2021 [21]	30	90	17/13	42/48	57.61 ± 4.16	56.91 ± 4.85	NA	ELISA	NA
Li XL, 2022 [29]	21	20	13/8	12/8	37 ± 5	38 ± 4	12 h, 24 h, and 48 h diagnosed with sepsis	NA	Retrospective study
Xiao HT, 2022 [30]	46	103	20/26	46/57	42.78 ± 8.75	40.26 ± 9.22	ICU admission	ELISA	NA
Yu DY, 2022 [31]	67	95	37/30	51/44	70.3 ± 8.3	69.7 ± 8.6	NA	ELISA	Retrospective study

*ELISA* enzyme-linked immunosorbent assay, *CLIA* chemiluminescence immunoassay, *ICA* immunochromatography assay, *SAE* sepsis-associated encephalopathy patients, *NE* no − encephalopathy septic patients, *NA* not announced, *h* hours, *ICU* intensive care unit

Yao B, et al. [16]

### Quality assessment

**Fig. 2:**
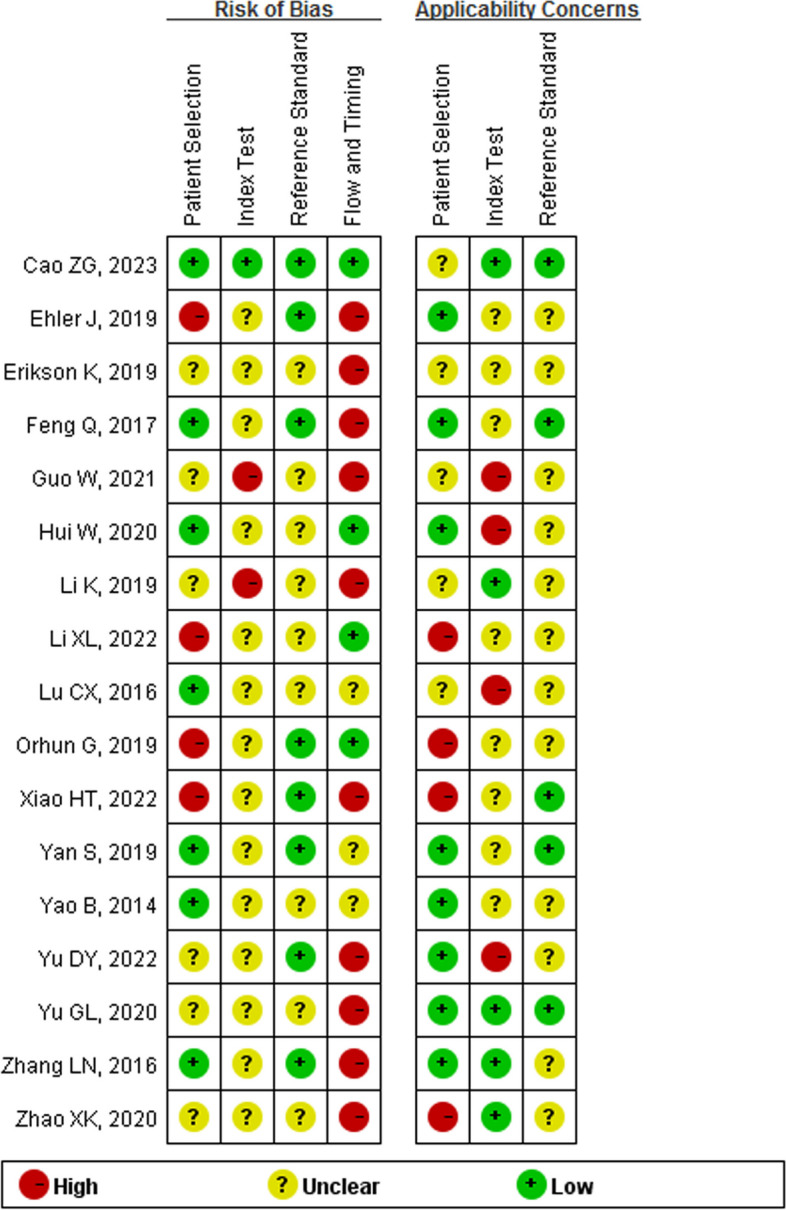
Separate summaries of risk bias and applicability concerns were presented for each study

**Fig. 3:**
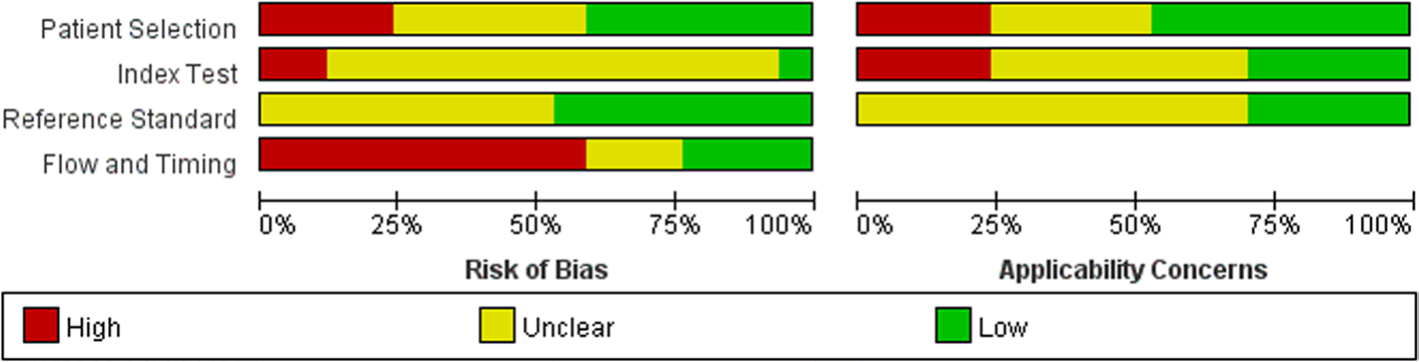
Risk of bias and applicability concerns in all included studies presented as percentages

QUADAS-2 was used to assess the risk of bias and applicability concerns (Figs. 2 and 3). As shown, a total of 7 studies, 1 study, 8 studies and 4 studies with low risk of patient selection, index test, reference standard, and flow and timing, respectively. There are 8 studies, 5 studies and 5 studies with low concern regarding of patient selection, index test and reference standard, respectively.

### *Meta*-analysis of serum NSE between SAE and NE patients

**Fig. 4:**
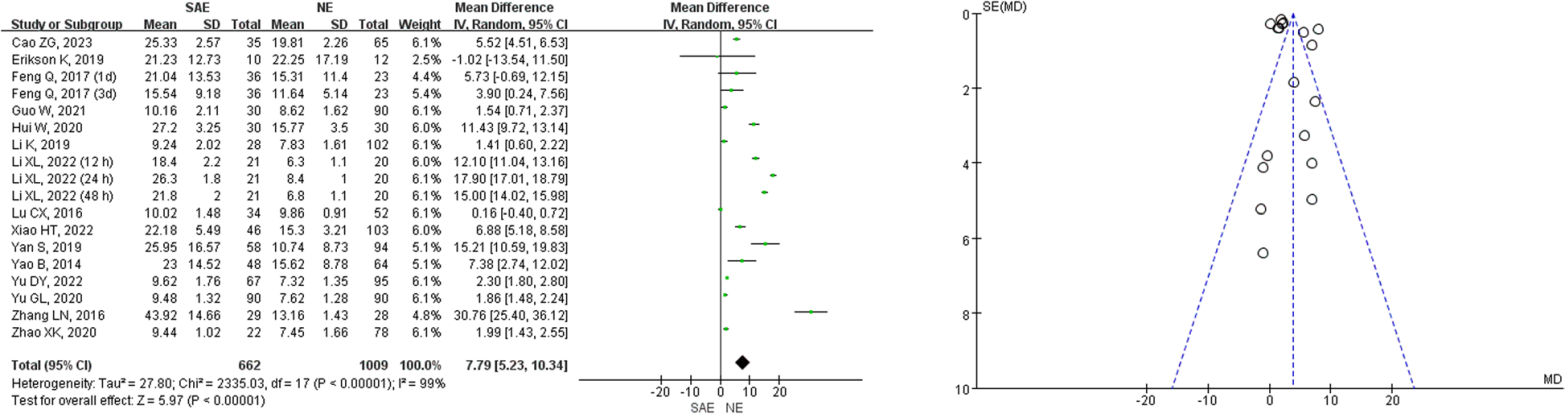
Meta-analysis of serum NSE between SAE and NE patients

Among the 17 studies included in this analysis, 15 of them compared serum NSE levels between SAE patients and NE patients (Fig. 4). All studies measured serum NSE levels in ng/mL, allowing us to use the mean difference (MD) to estimate the differences in serum NSE levels between SAE and NE patients. However, the results of the heterogeneity test showed a significant difference (*I*^2^ = 99%, *P* < 0.001). Hence, a REM was adopted for the meta-analysis, and results indicated that the serum NSE levels among SAE patients (*n* = 662) were significantly higher compared to those among NE patients (*n* = 1009) (*Z* = 5.97, *P* < 0.001, MD = 7.79, 95%CI 5.23–10.34).

### *Meta*-analysis of serum NSE in prognosis of SAE patients

**Fig. 5:**
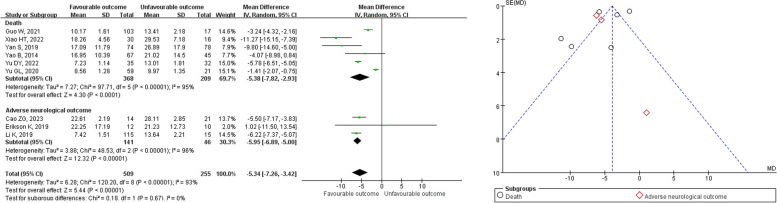
Meta-analysis of serum NSE in the prognosis of SAE patients

A total of 9 studies examined the prognosis of patients with SAE and NE (sepsis patients), with 6 studies focusing on mortality and 3 studies focusing on adverse neurological outcomes (Fig. 5). Firstly, the results of the subgroup differences test between the meta-analysis reporting mortality and the meta-analysis reporting adverse neurological outcomes indicated no heterogeneity (*I*^2^ = 0%, *P* = 0.67). However, heterogeneity tests showed significant differences in both the meta-analysis reporting mortality (*I*^2^ = 96%, *P* < 0.001) and the meta-analysis reporting adverse neurological outcomes (*I*^2^ = 93%, *P* < 0.001). Consequently, a REM was used for the meta-analysis. The findings suggested that the serum NSE levels in sepsis patients with a favorable outcome (*n* = 509) were significantly lower than those in sepsis patients with an unfavorable outcome (*n* = 255) (*Z* = 5.44, *P* < 0.001, MD = − 5.34, 95%CI − 7.26 to − 3.42) in both the meta-analysis reporting mortality (*Z* = 4.30, *P* < 0.001, MD = − 5.38, 95%CI − 7.28 to − 2.93) and the meta-analysis reporting adverse neurological outcomes (*Z* = 12.32, *P* < 0.001, MD = − 5.95, 95%CI − 6.89 to − 5.00).

### Meta-analysis of different assay methods for serum NSE between SAE and NE patients

**Fig. 6:**
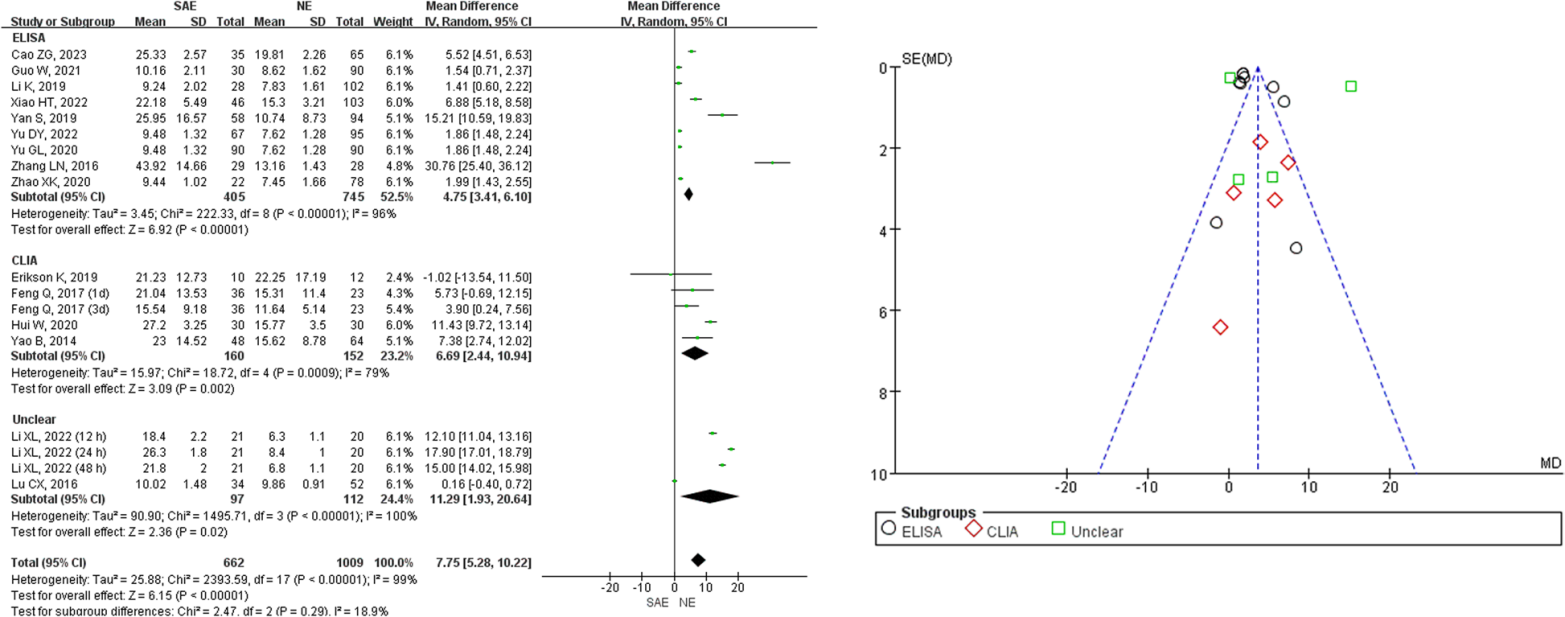
Meta-analysis of different assay methods for serum NSE between SAE and NE patients

Out of the 17 studies, 10 reported serum NSE levels based on enzyme-linked immunosorbent assay (ELISA), 5 reported serum NSE levels using sensitive automated chemiluminescent immunoassay (CLIA), and 2 did not report on the detection of serum NSE (Fig. 6). Firstly, the results of the subgroup differences test between the meta-analyses of different assay methods for serum NSE between SAE and NE patients suggested no heterogeneity (*I*^2^ = 18.9%, *P* = 0.29). However, heterogeneity tests showed significant differences in both the meta-analysis reporting the detection of serum NSE using ELISA (*I*^2^ = 96%, *P* < 0.001) and the meta-analysis reporting the detection of serum NSE using CLIA (*I*^2^ = 79%, *P* < 0.001), as well as the meta-analysis that did not report anything about the detection of serum NSE (*I*^2^ = 100%, *P* < 0.001). As a result, a REM was used for the meta-analysis. The findings indicated that the serum NSE levels in SAE patients (*n* = 662) were higher in contrast with those in NE patients (*n* = 1009) with statistical significance (*Z* = 6.15, *P* < 0.001, MD = 7.75, 95%CI 5.28–10.22) in both the meta-analysis reporting the detection of serum NSE using ELISA (*Z* = 6.92, *P* < 0.001, MD = 4.75, 95%CI 3.41–6.10), the meta-analysis reporting the detection of serum NSE using CLIA (*Z* = 3.09, *P* < 0.001, MD = 6.69, 95%CI 2.44 to 10.94), and the meta-analysis that did not report anything about the detection of serum NSE (*Z* = 2.36, *P* = 0.02, MD = 11.29, 95%CI 1.93 to 20.64).

### Meta-analysis of different study methods for serum NSE between SAE and NE patients

**Fig. 7:**
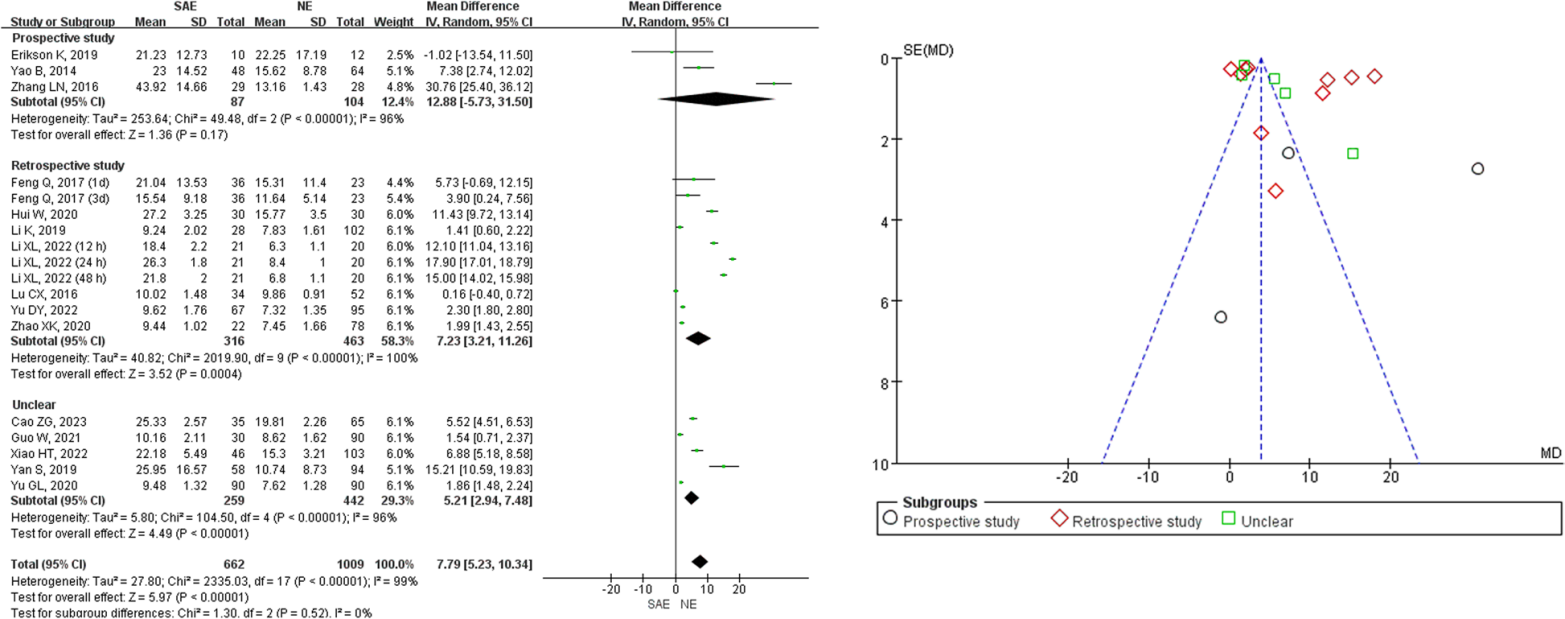
Meta-analysis of different study methods for serum NSE between SAE and NE patients

Out of the 17 studies, 5 reported a prospective study design, 10 reported a retrospective study design, and 5 did not provide information about their study methods (Fig. 7). The subgroup meta-analysis results indicated that the study methods were not sources of heterogeneity (*I*^2^ = 0%, *P* = 0.52). However, significant heterogeneity was observed in the subgroups reporting prospective studies (*I*^2^ = 96%, *P* < 0.001), retrospective studies (*I*^2^ = 100%, *P* < 0.001), and no information about study methods (*I*^2^ = 99%, *P* < 0.001). The combined meta-analysis results showed that the serum NSE levels among SAE patients (*n* = 662) were higher in contrast with those in NE patients (*n* = 1009) with statistical significance (*Z* = 5.97, *P* < 0.001, MD = 7.79, 95%CI 5.23–10.34) in both the meta-analysis reporting prospective studies (*Z* = 1.36, *P* = 0.17, MD = 12.88, 95%CI − 5.73–31.50), the meta-analysis reporting retrospective studies (*Z* = 3.52, *P* < 0.001, MD = 7.23, 95%CI 3.21–11.26), and the meta-analysis reporting no information about study methods *Z* = 4.49, *P* = 0.02, MD = 5.21, 95%CI 2.94–7.48).

## Discussion

This meta-analysis aimed to investigate the clinical value of serum NSE among SAE patients. SAE is a condition that arises from the systemic inflammatory response triggered by an infection in the patient's body, affecting various aspects such as neurotransmitter transmission, microcirculation, blood–brain barrier, and neuroinflammatory response. It is important to note that SAE is not a direct consequence of the infection itself. The main clinical manifestations observed in SAE patients include impaired consciousness, mild cognitive dysfunction, and delirium [32, 33]. The occurrence of SAE may be associated with increased levels of inflammatory cytokines, oxidative damage, mitochondrial dysfunction, and neuronal apoptosis [34, 35]. However, there has been currently no agreed definition of SAE or established biomarkers for its diagnosis. Clinicians rely on their own clinical experience and expertise to diagnose SAE due to this lack of consensus.

S100 calcium-binding protein B (S100B) and NSE are two proteins frequently mentioned in relation to brain diseases, such as brain injury, neurological dysfunction, or brain dysfunction. Elevated levels of these proteins in both serum and cerebrospinal fluid indicate the presence of brain injury [36–38]. A meta-analysis of 28 studies demonstrated that SAE patients had higher serum S100B levels in contrast with those with NE. Furthermore, it was observed that serum S100B was associated with prognosis in sepsis patients. These findings suggest that serum S100B could be a potential biomarker for diagnosing and predicting prognosis in patients with SAE, offering a means to assess brain injury in patients with sepsis [39]. However, the diagnostic and prognostic value of serum NSE in patients with SAE remains controversial, and no relevant meta-analyses have been published on this topic. Some studies have indicated that serum NSE is not as effective as S100B and the amino-terminal propeptide of the C-type natriuretic peptide (NT-proCNP) as a marker for diagnosis and prognosis in patients with SAE [16, 18]. Conversely, another study found that serum NSE performed similarly to serum S100B [22].

In the present study, the results of a meta-analysis suggested that serum levels of NSE were significantly higher compared to NE patients. Additionally, sepsis patients with a favorable outcome had significantly lower levels of serum NSE in contrast with those with an unfavorable outcome. A retrospective study found that SAE patients had higher levels of serum NSE, which exhibited a negative correlation with peripheral CD4 lymphocyte count (*r* = − 0.738, *P* < 0.01). Peripheral CD4 lymphocyte count was considered an independent diagnostic marker for septic encephalopathy [17]. Erikson K et al. discovered that septic shock patients with delirium had markedly higher serum NSE levels compared to septic shock patients without delirium [19]. Orhun G et al. observed significantly elevated serum NSE levels in sepsis-induced brain dysfunction (SIBD) patients in contrast with healthy individuals. Furthermore, they revealed lower serum NSE levels in SIBD patients with delirium in contrast with those with coma [20]. Additionally, serum NSE showed a significant association with several biomarkers previously identified as useful for diagnosis and prognostic prediction in patients with SAE, such as miR-29a [21]. Based on these above studies, combined with our meta-analysis results, it could be inferred that NSE levels are elevated in the serum of patients with SAE.

Although many studies have demonstrated the potential of serum NSE as a prognostic indicator for patients with SAE [16, 39], its sensitivity and specificity remain obscure, mainly because other disorders such as shock [40], hemolysis [41, 42], lung cancer [43, 44], and prostate cancer [45, 46] can also cause elevated serum NSE levels. Out of the 17 studies included in the present analysis, 6 studies provided thresholds, sensitivities, and specificities for the diagnosis or prognosis of SAE [11, 16, 22, 23, 30, 31]. Yao B et al. discovered that a serum NSE level of 24.145 ng/mL had a specificity of 82.8% as well as a sensitivity of 54.2% for diagnosing SAE. Additionally, a level of 24.865 ng/mL predicted hospital mortality with a specificity of 79.1% and a sensitivity of 46.7% [16]. Zhang LN et al. reported that a serum NSE level of 0.79 ng/mL had a specificity of 87.5% and a sensitivity of 58.30% for diagnosing SAE. Furthermore, a level of 27.02 ng/mL had a specificity of 88.90% as well as a sensitivity of 60.00% for diagnosing 28-day mortality in SAE [11]. Cao ZG et al. reported that serum NSE levels had a sensitivity of 79.03% as well as a specificity of 83.29% for diagnosing SAE [22].

However, in this meta-analysis, we discovered undeniable heterogeneity among the studies due to factors such as age, gender, timing of sample collection, primary disease, and therapeutic drugs, et al. Additionally, there was a potential risk of publication bias that may have magnified the correlation of NSE with the diagnosis and prognosis of SAE. Furthermore, we were unable to assess the diagnostic power of serum NSE due to the unavailability of complete data regarding its role in the diagnosis and prognosis of SAE.

### Limitations

This meta-analysis had some limitations. (1) Due to a lack of data disclosure, we were unable to conduct a sensitivity analysis, meta-regression analysis, and utilize the GRADE approach. (2) Ethnic differences were also not considered in this meta-analysis. (3) The majority of the studies included in our analysis were carried out at single centers, which imposed the limitations associated with such studies on our meta-analysis results.

### Future directions

The results of our meta-analysis suggested that serum NSE levels were related to the development of SAE patients and their prognosis. However, there was no evidence to indicate whether serum NSE could be utilized as a basis for clinical treatment to improve the prognosis of SAE patients. Additionally, it remains unclear whether serum NSE levels differ among different races.

## Conclusion

The level of serum NSE in SAE patients was higher in contrast with that in NE patients. Higher serum NSE levels were related to an unfavorable outcome among sepsis patients.

## Data Availability

The original contributions presented in the study are included in the article/Supplementary material, further inquiries can be directed to the corresponding author.
